# Functional, Nutraceutical and Health Endorsing Perspectives of Saffron

**DOI:** 10.1002/fsn3.70721

**Published:** 2025-08-06

**Authors:** Tabussam Tufail, Huma Badr ul Ain, Ali Ikram, Muhammad Tayyab Arshad, Muhammed Adem Abdullahi

**Affiliations:** ^1^ College of Pharmaceutical Science Zhejiang University of Technology Hangzhou People's Republic of China; ^2^ University Institute of Diet and Nutritional Sciences The University of Lahore Lahore Pakistan; ^3^ School of Food Science and Engineering Yangzhou University Yangzhou China; ^4^ University Institute of Food Science and Technology The University of Lahore Lahore Pakistan; ^5^ Functional Food and Nutrition Program Faculty of Agro‐Industry, Prince of Songkla University Songkhla Thailand; ^6^ Department of Food Science and Postharvest Technology Jimma University College of Agriculture and Veterinary Medicine, Jimma University Jimma Ethiopia

**Keywords:** bioactive components, *Crocus sativus*, saffron, spice

## Abstract

*Crocus sativus* L., widely recognized as saffron, is a high‐value spice renowned for its culinary and medicinal applications. Cultivated primarily in regions like Iran, Greece, and India, saffron is derived from the red stigmas of the Crocus flower. Saffron's distinct aroma and therapeutic properties stem from its rich composition of volatile compounds, including terpene alcohols, terpenes, and their esters. Extensive research has highlighted saffron's diverse pharmacological effects, establishing its role as a potent agent in both traditional and modern medicine. Its bioactive components exhibit significant antioxidant, antimicrobial, anti‐inflammatory, and antinociceptive activities. Furthermore, saffron has demonstrated promising potential in treating various conditions due to its anticonvulsant, antidepressant, antibacterial, antitumor, anticancer, antiaging, and antidiabetic properties. This review provides an in‐depth analysis of saffron's chemical composition and wide‐ranging health benefits. It explores the therapeutic potential of saffron in managing conditions such as neurodegenerative disorders (e.g., Alzheimer's and Parkinson's diseases), erectile dysfunction, cardiovascular diseases, liver diseases, pancreatic disorders, primary dysmenorrhea, gastrointestinal disorders (e.g., gastritis, peptic ulcers), and skin‐related disorders. While saffron presents significant therapeutic promise, it is crucial to consider its toxicological profile. Although generally regarded as safe for human consumption in moderate quantities, high doses of saffron can lead to adverse effects. This review offers a comprehensive overview of saffron's bioactive constituents, therapeutic potential, and safety profile, emphasizing its significance as a multifaceted natural product with applications in food and medicine.

## Graphical abstract

Saffron is generally regarded as safe for human consumption in moderate quantities, its potential adverse effects at higher doses require careful consideration. This review presents a balanced perspective on saffron's medicinal potential and its safe usage, making it a valuable resource for researchers and healthcare professionals.

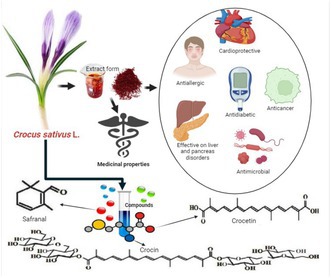

## 1 Introduction

Saffron, also known as *Crocus sativus* L., is a popular herbal remedy widely grown in India, Iran, and the Mediterranean. It has a variety of uses in traditional medicine, and it is a popular spice in cooking. Only in the fall does the Iridaceae plant *C. sativus* flower; it is dormant in the summer. Saffron, the name of its stigma and a derivative of the Arabic word Azafran, is one of the costly spices (Rahmani et al. 2019). Biologically speaking, *C. sativus* has petals that range in color from violet to mauve. 20–30 cm tall, *C. sativus* is covered by 5–11 nonphotosynthetic, white leaves (cataphylls) and has 5–11 true leaves that are protected. In October, the shrub blooms with purple stripes and a honey‐like aroma (Kouchaki et al. 2006).

The flower of saffron contains 0.74% ash, 1.07% protein, 6.12% carbohydrate, and 0.32% lipids. Stamens had the highest percentage of mineral content (Serrano‐Díaz et al. 2013). Because of its therapeutic properties, saffron consumption increases daily. With the increased demand for saffron, farmers in various countries worldwide have begun cultivating *C. sativus* . Multiple studies have highlighted the function of saffron in treating various conditions. Saffron's health‐promoting properties can also be found in ancient Chinese, Unani, Ayurvedic, and Homeopathic prescriptions (Abu‐Izneid et al. 2022).

*Crocus sativus* has been studied for its ability to act as an adaptogen, CNS depressant, emmenagogue agent, and anti‐asthmatic (Gohari et al. 2013). Active substances such as safranal, picrocrocin, crocetin, and crocin can be found in variable amounts in the tepals, stigmas, leaves, and corns of the *C. sativus* plant. These bioactive components have been demonstrated to improve health by modulating various physiological and biological processes. Findings from a few previous studies show that saffron is safe to consume at multiple tested levels and has no adverse side effects. Although saffron has traditionally been used as a spice, the literature supports its use in ethnomedicine (Butnariu et al. 2022).

Saffron is used in ethnomedicine to cure conditions such as constipation, mucous inflammation, depression, lung congestion, hunger stimulation, coughing, menstrual flow stimulation, lactation enhancement, and cramping. Saffron has been utilized as an emmenagogue, adaptogenic, and expectorant in Ayurvedic medicine (Gohari et al. 2013). Erysipelas has been treated with saffron in conventional Persian medicine. Greeks utilized saffron to heal acne, wounds, and many other skin conditions (Mollazadeh et al. 2015). Organic dyes derived from animals, minerals, and plants have been used since the Stone Age. Yellow and saffron dyes made from saffron have been created for painting and coloring garments (Yildirim et al. 2020).

The main active ingredients in saffron are crocin, picrocrocin, crocetin, and safranal, which have several advantageous effects, including antioxidant activity, analgesia, cardiovascular protection, and suppression of cancer, inflammation, diabetes, and depression (Yang et al. 2018). In China, India, and Spain, saffron and the mentioned ingredients are frequently given to prevent or cure cardiovascular disease, liver illness, reproductive issues, and type 2 diabetes mellitus (Pourmasoumi et al. 2019). The stigma component of saffron has been linked to the spice's pharmacological qualities, which include anticancerous, hepatotoxic‐disease protection, anti‐inflammatory, antioxidant, pro‐apoptotic, and antidepressant effects (Bathaie et al. 2011).

Moreover, saffron byproducts can be used as sources of organic fertilizer and added value as food additions, and comply with the principles of the circular economy (Tuberoso et al. 2016). Because they have a low prevalence of adverse effects, medicinal plants are regarded as one of the numerous essential and effective methods for health intervention. Over 300 volatile and nonvolatile metabolites, such as crocin, picrocrocin, safranal, monoterpenes, aldehydes, and several other carotenoids with therapeutic properties, are found in saffron (Winterhalter and Straubinger 2000).

This review aims to thoroughly analyze saffron by examining its pharmacological characteristics, chemical makeup, and range of traditional and modern medical applications. Among the goals are assessing saffron's potential for medicinal application, determining any potential toxicological risks related to its use, and investigating its functional and nutritional qualities. The review aims to provide insights into the potential of saffron as a functional food or supplement while highlighting its advantages and disadvantages.

## 2 Phytochemistry of Saffron

**FIGURE 1:**
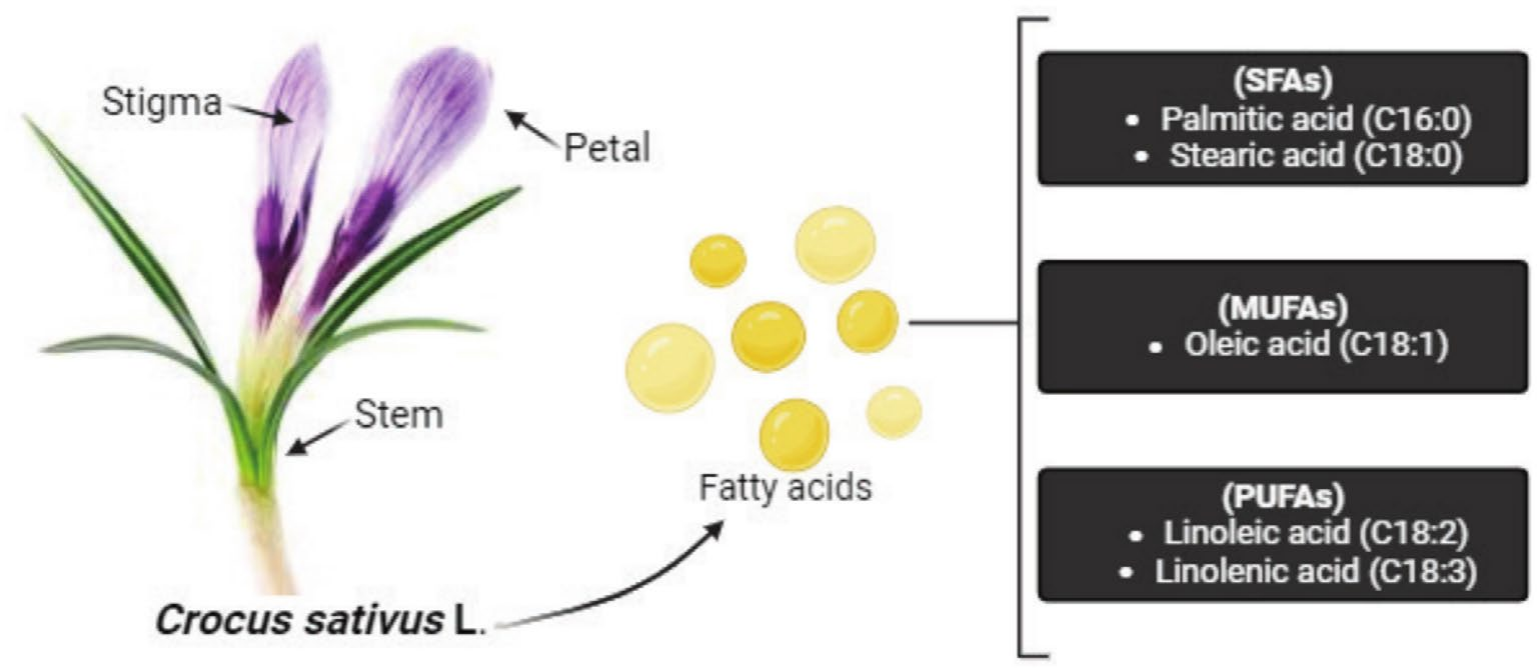
Fatty acid composition in different parts of saffron.

Saffron's main ingredients include water 14%–16%, nitrogenous matter 11%–13%, sugars 12%–15%, soluble extract 4%–44%, volatile oil 0.6%–0.9%, fiber 4%–5%, and total ash 4%–6%. Riboflavin, thiamin, and a trace amount of beta‐carotene are essential vitamins in saffron (Table 1). The vitamin with the highest concentration in saffron, ranging from 56 to 138 ng/g, is riboflavin. Between 0.7 and 4 g/g of thiamine can be found in saffron. The petroleum ether extract from bulbs has also been discovered to contain essential fatty acids, including linoleic and linolenic (Christodoulou et al. 2015; Figure 1).

**TABLE 1:** Nutritional composition of saffron per 100 g (Haytowitz et al. 2011).

Nutrient	Value	RDA (%)
Energy	310 Kcal	15.5
Carbohydrates	65.37 g	50
Dietary fiber	3.9 g	10
Protein	11.43 g	21
Total fat	5.85 g	29
Niacin	1.46 mg	95
Vitamin A	530 IU	18
Vitamin C	80.8 mg	90
Thiamin	0.115 mg	10
Pyridoxine	1.010 mg	77
Riboflavin	0.267 mg	20
Folates	93 mcg	23
Potassium	1724 mg	37
Sodium	148 mg	10
Calcium	111 mg	11
Zinc	1.09 mg	10
Phosphorus	252 mg	36
Manganese	28.408 mg	1235
Magnesium	264 mg	66
Iron	11.10 mg	139

Sterols (campesterol, stigmasterol, and beta‐sitosterol), ursolic, palmitoleic, oleanolic, and oleic acids are also contained in saffron. Volatile, nonvolatile, and aromatic chemicals abound in saffron. These substances consist of traces of hydrophobic and hydrophilic proteins, carbohydrates, minerals, vitamins, gums, pigments, mucilage, saponins, alkaloids, crocins, safranal, picrocrocin, and other substances (Kosar et al. 2017).

Crocetin and its related glucosidic derivatives are the main constituents of saffron that give the stigmatic lobes of the spice their scarlet color (Ghanbari, Khajoei‐Nejad, Erasmus and van Ruth 2019). But carotenoids (crocetin) in the form of crocetin, which also comprises diglucoside, gamma‐crocetin, beta‐carotene, and zeaxanthin as glycosidic forms, are the primary components of saffron. Picrocrocin is the primary element in saffron that gives it a bitter flavor. It crystallizes after hydrolysis, yielding safranal and glucose (Table 2; Zouagui and Sbabou 2022).

**TABLE 2:** Different parts of saffron, their bioactive components and health benefits.

Parts	Bioactive components	Benefits	References
Stigmas	Safranal, picrocrocin, crocin, crocetin, and phenolic acids	It reduces oxidative stress by neutralizing free radicals and relieves mild to moderate depression and elevates mood It guards against Parkinson's and Alzheimer's diseases It stops the growth of tumors and causes cancer cells to die. It enhances lipid profile and lowers blood pressure It reduces the risk of chronic illness and inflammation It guards against AMD or age‐related macular degeneration, increases sexual desire and treats erectile dysfunction	Singletary (2020)
Petals	Anthocyanins, saponins, and flavonoids (kaempferol, quercetin)	Like stigmas, it prevents oxidative damage and has mood‐enhancing qualities. Potential to prevent cancer cells from proliferating It helps the body heal from wounds and restore damaged tissue by lowering inflammatory indicators	Ghasemzadeh Rahbardar and Hosseinzadeh (2023)
Corms	Saponins, alkaloids, and polysaccharides	It enhances insulin sensitivity, helps control blood sugar levels, and shields cells from oxidative damage It balances immunological responses, lowers inflammation, promotes gut and joint health and bolsters immune system performance	wang et al. 2022; Ghanbari, Khajoei‐Nejad, Van Ruth and Aghighi (2019)
Leaves	Carotenoids, phenolic compounds, and flavonoids	It lowers chronic inflammation and offers defense against oxidative stress It could improve digestion and enhance intestinal health	Marrone et al. (2024)

## 3 Phytochemical Profile of Saffron

### 3.1 Apocarotenoids

Apocarotenoids are produced through oxidative cleavage of the main carotenoids by oxygenases known as carotenoid cleavage dioxygenases (CCDs). CCDs cleave one or two double bonds in the carotenoids by identifying the double bonds, producing apocarotenoids (Gohari et al. 2013). Saffron's main byproducts include crocin, picrocrocin, safranal, and crocetin. There are tons of crocins in the genus Crocus. C_44_ H_70_ O_28_ is the chemical formula with a molecular weight of 976.96 g/mol (Seifi and Shayesteh 2020).

Crocin is quite an odd mixture of carotenoids with cis and trans‐diesters that is extensively used as a coloring ingredient in various pharmaceuticals and foods (Pandita 2021). The trans‐crocetin di‐D gentiobiosyl compound, known as crocin, gives saffron its orange‐yellow color. Numerous crocin variants exist, including trans‐crocin 4, trans‐crocin 3, trans‐crocin 2, crocetin, cis‐crocin 4, and tricrocin, among others (Ahrazem et al. 2015).

The monoglycosyl polyene or diglycosyl polyene ester of crocetin is known as crocin. Because crocin contains esterified gentiobiose, it is the ideal coloring agent for various foods and cuisines. Unusual olefin conjugate crocetin has a molecular weight of 328.4 g/mol, a melting point of 285°C, and is classed as a lipophilic carotenoid. The degradation and cleavage of zeaxanthin on 8′,8/7 double bonds result in the formation of various kinds of crocetins, including crocetin‐I, a‐crocetin, g‐crocetin, and crocetin‐II (Patel and Boghra 2018).

According to many experts, 94% of the crocetin in saffron is found as glycosides, whereas 6% is found as free crocetin (Giaccio 2004). Oil‐soluble and hydrophobic by nature, crocetin is a conjugated polyene dicarboxylic acid that amplifies the saffron's distinctively pleasing scent. Despite not possessing the provitamin function, crocetin is a member of the wide family of natural colors known as carotenoids. However, a tiny subset of carotenoids cannot be incorporated into the overall chemical structure because they include acid and carboxylic groups (Caballero‐Ortega et al. 2004). These few compounds include crocetin and 8,8‐diapo8,8 caroteneic acid. It comprises four methyl groups, seven double bonds, and a diterpenic, symmetrical structure. Its molecular weight is 328.4 and its primary structure is C_20_H_24_O_4_. Crocetin is a chemical compound with a melting point of 285°C and crystallizes in the form of red needles, though it appears yellow in solution. It is only moderately soluble in water (20 mM at pH 8) but highly soluble in organic bases like pyridine. Exceeding its solubility in water leads to a yellow precipitate (Giaccio 2004).

Crocetin is well known for its antioxidant properties and is an essential component of saffron. The bitter glucoside picrocrocin, with a chemical formula of C_16_H_26_O_7_ and a molecular weight of 330.37 g/mol, gives saffron flavor. The precursor compound for safranal, picrocrocin, is a colorless glycoside. It is a monoterpene chemical and the second‐most crucial component of saffron (Wani et al. 2022). Conversely, safranal has a molecular weight of 150.21 g/mol and is chemically a monoterpene aldehyde and a picrocrocin glycon. Iranian saffron samples were analyzed using GCMS, and the results revealed many aromatic active chemicals, including safranal, dihydro‐oxophorone, and 4‐ketoisophorone (Jalali‐Heravi et al. 2009).

The saffron stigma contains apocarotenoids, although their presence is tightly controlled. As the red stigmas grow, the apocarotenoids' accumulation reaches its highest value. Five genes have been researched that are associated with the biosynthetic pathway of carotenoids, the expression of related genes, and cleavage activities (Javan and Hosseinzadehgonabad 2017). When saffron is dried after being harvested, picrocrocin is broken down into glucose and safranal. The chemical formula for safranal is C_10_H_14_O and its IUPAC name is cyclic terpene aldehyde 2,6,6‐trimethyl‐1,3trimethyl‐1,3‐cyclohexadiene‐1‐carboxaldehyde (Hosseinzadeh and Nassiri‐Asl 2013).

The volatile oil safranal produces saffron's characteristic aroma. Up to 70% of saffron's volatile component may be made up of safranal. There is no distinguishing scent to the newly plucked stigma. Saffron is treated and dried once it is gathered. D‐glucose and a free safranal molecule are produced when heat and enzymes are applied to picrocrocin. The spice's unique aroma is provided by the resultant safranal (Caballero‐Ortega et al. 2004).

### 3.2 Flavonoids

**FIGURE 2:**
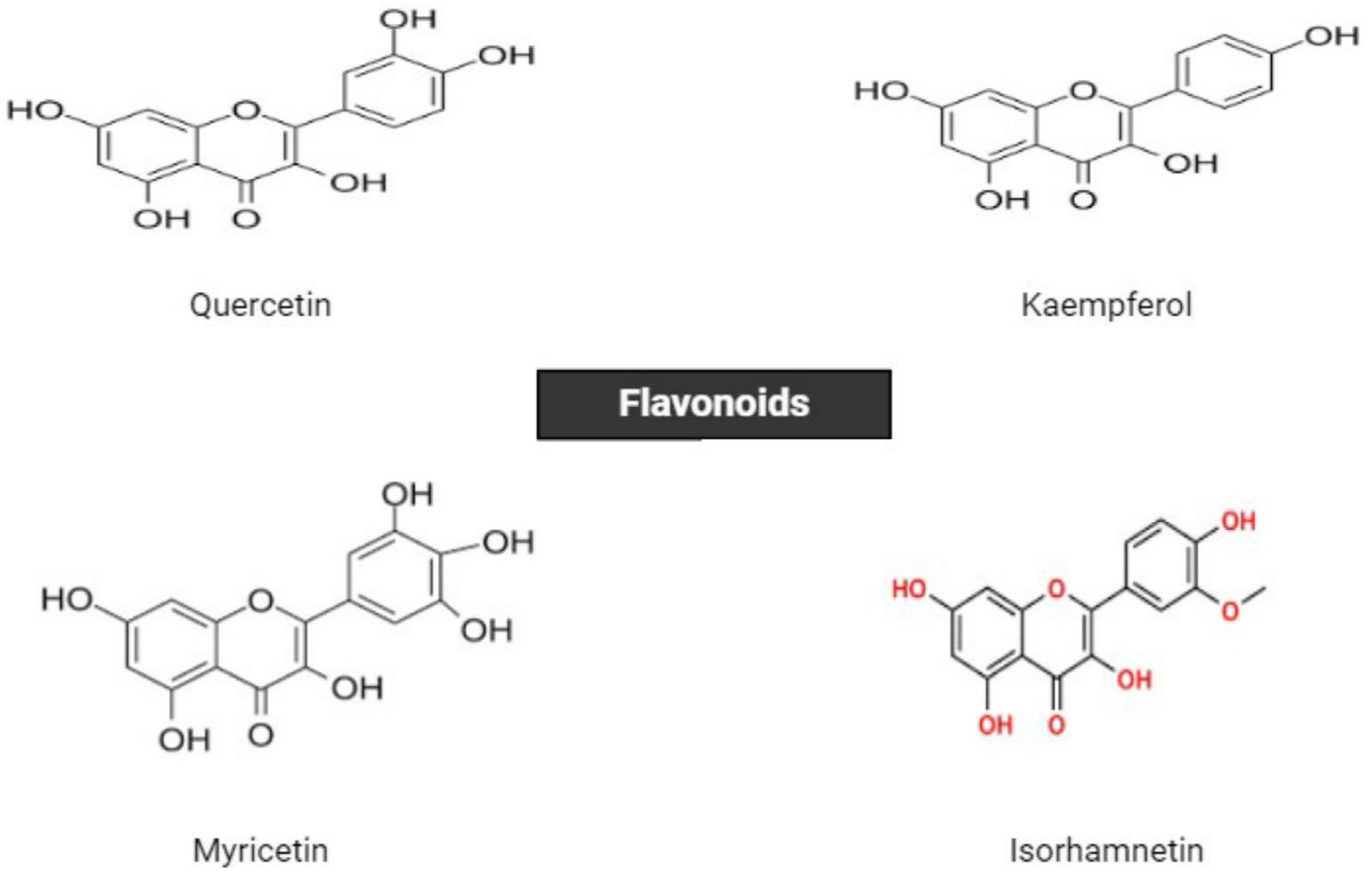
Chemical structure of flavonoid compounds in saffron.

In *C. sativus* , flavonols at the 3,7 and 4‐OH sites are glycosylated, creating a strand of flavonols (Karimi et al. 2010). Three kaempferol‐related glucosides have been identified: kaempferol 3‐O sophoroside 7‐O glucopyranoside (K3OS7OG), kaempferol 3,7,4 triglucoside (K374T), and kaempferol 7‐O sophoroside (K7OS) (Carmona et al. 2007). As the stigma of *C. sativus* develops, each of them grows. The most significant relative quantitative levels of K7OS are attained at the moment of anthesis, but the highest levels of K3OS7OG and K374T are reached during the scarlet stage, also known as the scarlet stage (Figure 2; Dhar et al. 2017).

Galangin, quercetin, and kaempferol have been isolated from fresh saffron petals, while gallic acid and pyrogallol have been discovered in the spice's stigma. Certain flavonoids found in ethyl acetate, diethyl ether, and some aqueous fractions include taxifolin 7‐O hexose (T7OH), 4‐methyl ether dihydrokaempferol 3‐O deoxy hexoside (4MEDK3ODH), and naringenin 7‐O hexoside (N7OH). The new flavonoids were found using methanolic extract (Trapero et al. 2012).

The extracted flavonoids primarily consist of kaempferol, quercetin glycosidic derivatives, and acetylated and methoxylated derivatives. Tepals have identified and described nearly 21 different taxifolin, kaempferol, naringenin, and isorhamnetin. Beta D‐glucopyranoside and kaempferol‐3‐O–glucopyranosyl‐1,2 are the two main tepal flavonoids (K3ODGP). The tepals are abundant in petunidin, anthocyanins, delphinidin, and malvidin, in addition to flavonoids (Mottaghipisheh et al. 2020).

### 3.3 Phenolic Acids

Caffeic acid, gallic acid, methylparaben, chlorogenic acid, and pyrogallol are particular hydroxycinnamic acids extracted and discovered in saffron (Table 3). It has been observed that several sections of *C. sativus* contain hydroxybenzoic acids, which are the building blocks for the manufacture of flavonoids. Vanillic acid, sinapic acid, and p‐hydroxybenzoic acid are among the hydroxycinnamic acids present in saffron petals, while benzoic acid and p‐hydroxybenzoic acid are also present in *C. sativus* pollens (Karimi et al. 2010).

**TABLE 3:** Phenolic acids in saffron and their effects.

Acids	Effects	References
Gallic acid	A phenolic acid prevalent in the plant kingdom exhibits potent antioxidant and anti‐inflammatory properties. Its presence in saffron is noteworthy, as it is believed to neutralize free radicals, potentially contributing to the spice's recognized anticancer and antimicrobial effects	Salamat et al. (2024)
Caffeic acid	Recognized for its antioxidant and anti‐inflammatory properties, it may mitigate oxidative stress and potentially inhibit carcinogenesis. Furthermore, it may offer cardioprotective benefits by enhancing endothelial function	Ege et al. (2020)
p‐coumaric acid	Ferulic acid, a phenolic compound, exhibits antioxidant properties that effectively mitigate lipid peroxidation, thereby protecting cellular structures from oxidative stress. Furthermore, research suggests potential anti‐inflammatory and anticancer effects associated with this compound	Azghandi Fardaghi et al. (2021)
Chlorogenic acid	Chlorogenic acid, a potent antioxidant in saffron, exhibits anti‐inflammatory properties and may enhance glucose metabolism, contributing to antidiabetic effects. Furthermore, it plays a protective role in liver and cardiovascular health	Saeed Alkaltham et al. (2021)
Ferulic acid	Ferulic acid, a phenolic compound, is recognized for its antioxidant, anticancer, and anti‐inflammatory properties. Its presence in saffron may contribute to the spice's neuroprotective qualities, potentially by mitigating oxidative stress and inflammation within neurons	Turcov et al. (2022)
Protocatechuic acid	Crocetin, a phenolic acid renowned for its antioxidant, anti‐inflammatory, and anticancer properties, may contribute to the comprehensive protective effects of saffron on various tissues, including the brain, liver, and cardiovascular system	Senizza et al. (2019)
Vanillic acid	Vanillic acid, a phenolic acid with antioxidant properties, is a significant component of saffron. Its presence is believed to contribute to the spice's anti‐inflammatory effects and potential to mitigate oxidative cell damage	Ege et al. (2020)
Syringic acid	Syringic acid, a naturally occurring phenolic compound, has garnered attention for its notable antioxidant and anti‐inflammatory properties. Preliminary research suggests that syringic acid may mitigate oxidative stress at the cellular level, potentially contributing to the protective effects of saffron against chronic diseases	Esmaeili et al. (2011)

### 3.4 Monoterpenoids

Picrocrocin (C_16_H_26_O_7_; MW: 330.37 g/mol) and safranal (C_10_H_14_O; MW: 150.21 g/mol) are the two main bioactive phytoconstituents found in saffron that are formed from the breakdown of zeaxanthin. Among these, picrocrocin is responsible for flavor and bitterness, and safranal gives saffron its distinctive smell. The saffron contains essential oil with picrocrocin, a monoterpene glycoside precursor to safranal. Saffron's vital oil production can range from 0.4% to 1.3% (Kanakis et al. 2007).

The second‐most important phytochemical in saffron's essential oil, it contributes roughly 1% to 13% of the dry weight of the spice and gives it a bitter flavor and taste (Samarghandian et al. 2014). The monoterpene aldehyde safranal, an aglycon of picrocrocin, is found in saffron's essential oil. Fresh stigmas of *C. sativus* lack safranal content; b‐glucosidase acts on picrocrocin, its precursor, under postharvest dehydration and storage conditions to create safranal. Few saffron samples have shown concentrations of safranal up to 70% of the total volatile content. Today, saffron's safranal content is frequently analyzed quantitatively and qualitatively using spectrophotometric and gas chromatographic methods (Maggi et al. 2009).

### 3.5 Saponins and Miscellaneous Compounds of Saffron

Numerous plants and plant‐based products contain large amounts of saponins. These are terpenoids in terms of their chemical makeup and have a variety of bioactivities, including antimicrobial, antioxidant, allelopathic, herbicidal, and insecticidal qualities. Because surfactants can produce foam, saponin is derived from the Latin word soap (Rubio‐Moraga et al. 2013).

The saponins are divided into triterpenoid and steroidal saponins based on their chemical makeup. Two saponins of a triterpenic type, namely azafrine‐1 and azafrine‐2, have been identified in *C. sativus* . Chemically speaking, prosapogenin is composed of 3‐O‐D‐glucopyranose iduronic echinocystic acid. Azafrine‐1 and azafrine‐2 are vital for the plant's defensive system since they are found in the outer portion of corms. Salicylic acid, catechol, cinnamic acid, vanillin, gentisic acid, caffeic acid, and syringic acid are other phenolic substances detected in saffron corms. Saffron corms include hexadecanoic acid, palmitic acid, octadecadienoic acid, pentadecane, eicosane, and other volatile chemicals (Keller et al. 2019; Wani et al. 2022).

## 4 Nutraceutical Potential of Saffron

### 4.1 Medicine

Oxidative stress is widely believed to be implicated in the pathogenesis of numerous chronic diseases and conditions. This phenomenon arises when a disparity exists between the production of reactive oxygen species (ROS) and the body's endogenous antioxidant defense mechanisms. ROS can inflict damage at the cellular level, culminating in inflammation and other deleterious effects that may contribute to the development of these diseases over time (José Bagur et al. 2017).

Saffron's potent antioxidant action, attributed to its crocins, crocetin, and safranal components, is undoubtedly related to its therapeutic properties, and we examined the effects of various concentrations of aqueous and ethanolic stigma extracts on the pain threshold of mice to determine their anti‐inflammatory efficiency. Both stigma extracts showed considerable anti‐acute inflammatory efficacy at higher doses (Ashktorab et al. 2019).

Moradzadeh et al. (2019) summed up the antileukemic actions of saffron as slowing the proliferation of cancer cells by preventing the production of nucleic acids, boosting the immune system, and triggering promyelocytic leukemia differentiation at low doses (10 M). Saffron was given to participants in an 8‐week clinical research who had metabolic syndrome, a collection of cardiovascular risk factors including elevated lipid profiles, low HDL, high fasting blood sugar (FBG), hypertension, and abdominal obesity. The findings demonstrated a correlation between crocin supplements, a considerable rise in blood CETP concentration and HDL levels, and a decrease in cholesterol (Ghaffari and Roshanravan 2019).

The pharmacological effects of saffron on many gastrointestinal tract organs, including the liver, pancreas, ileum, and colon, were documented by Khorasany and Hosseinzadeh (2016). In rats with streptozotocin‐induced diabetes, Rahbani et al. (2012) examined the hepatoprotective effects of ethanolic saffron extracts. This study showed that saffron's antioxidant potential helped serum indicators of liver tissue injury and reduced diabetic hepatopathy. Concerning type 2 diabetes, according to Yaribeygi et al. (2019) saffron has powerful hypoglycemic effects through the promotion of insulin sensitivity, enhancement of cell function through inhibition of harmful pathways involved in cell failure that result in insufficient insulin release, and induction of GLUT4 translocation into the plasma membrane from the intracellular pool, which increases glucose uptake by insulin‐dependent tissues. Saffron‐enriched rye bread can be utilized in diabetes therapy since it increases insulin secretion and lowers triglyceride levels. Saffron has been studied for its potential benefits in treating various eye conditions, and some early studies have shown promising results. However, while these findings support the potential therapeutic effects of saffron, it is important to note that more long‐term safety data and large‐scale clinical trials are needed to fully understand its potential as a therapeutic agent and establish safe and effective dosages (Broadhead et al. 2019).

Additionally, it is important to note that not all studies have produced consistent results, and more research is needed to fully understand the mechanisms of action of saffron in the treatment of eye conditions. Spice's risk profile as a medicinal agent observed that saffron therapies with dosages of 100 and 200 mg/kg significantly reduced lipid peroxidation and brain nitric oxide levels as compared to the I/R group that was not treated in their study of the role of vascular endothelial growth component in the neuroprotective effects of saffron against cerebral hypoxia injury (I/R) in rats (Ramadan et al. 2012).

Saffron and its metabolites, notably crocetin esters, have been demonstrated to have favorable pharmacological impacts on the central nervous system, including biological activities on memory and learning, anxiety, and distress. Memory and learning abnormalities are typically associated with neurodegenerative disorders (Linardaki et al. 2017). Patients with multiple sclerosis who received a 7‐day course of saffron extract therapy showed improvement in oxidative stress in the hippocampus as well as deficits in spatial learning and memory caused by ethidium bromide. Furthermore, rats treated with ethanolic extracts of saffron at 5 g/rat and 10 g/rat had lower antioxidant potential (FRAP value) and TBARS levels (a marker of lipid peroxidation) (Ghaffari et al. 2015).

The antidepressant qualities of saffron have been extensively researched, and it has been advocated as a potentially useful and well‐tolerated therapy for depression and anxiety (Ghaffari and Roshanravan 2019). Curcumin (a chemical found in the spice turmeric) and a combination of curcumin/saffron therapy were useful in decreasing depressive and anxiolytic symptoms in individuals with major depressive disorder for 12 weeks (Shafiee et al. 2018). According to research, saffron (15 mg, 2 capsules per day) ranges of health in safe and effective treatment of mild to moderate depression with concomitant anxiety throughout a 6‐week trial period (Omidkhoda and Hosseinzadeh 2022).

Saffron has also been linked to anticonvulsant, sedative, antihistamine, anti‐asthmatic, and anti‐sexual dysfunction properties. The hazardous effects of saffron extracts must be evaluated before a therapy can be declared safe. Saffron is not harmful to humans in doses less than 1.5 g, but it is hazardous in doses greater than 5 g (which has an abortive effect) and can be lethal in doses of up to 20 g per day (Mzabri et al. 2019).

### 4.2 Food

**FIGURE 3:**
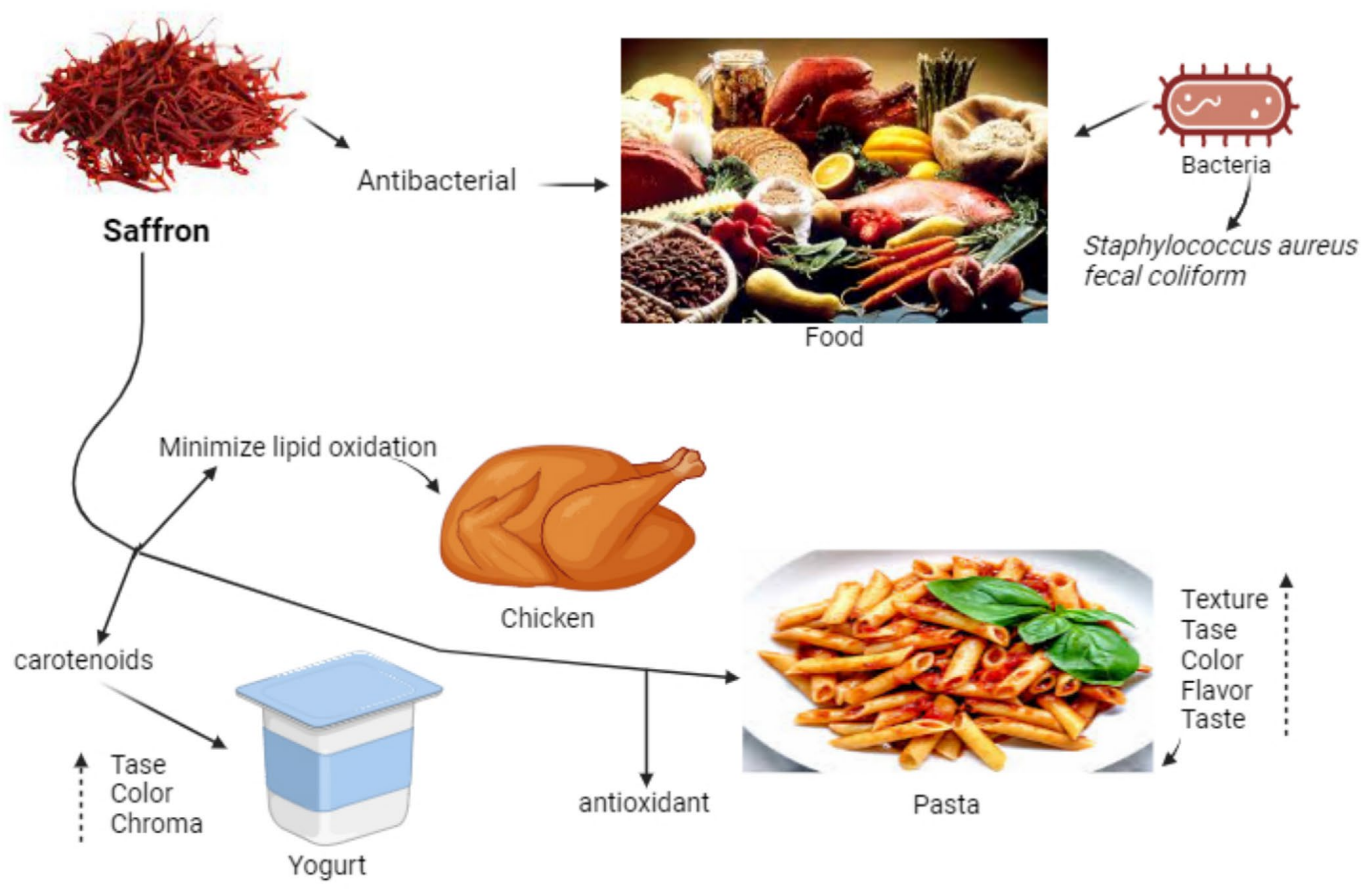
Applications of saffron in different food items and impact on taste, color, and preservation.

*Salmonella enterica* is a harmful bacterium that contaminates food and investigates saffron's antibacterial activity against this sickness. The bactericidal and inhibitory concentrations of safranal and crocin were 8–16 mg mL^−1^ and 64–128 mg mL^−1^, respectively (Pintado et al. 2011). Masoumi et al. (2018) discovered that saffron improves physicochemical properties by minimizing lipid oxidation in chicken breast meat during storage. Still, it does not affect microbiological development ( *Staphylococcus aureus* , *fecal coliform*) (Figure 3).

According to studies, higher saffron concentrations (0.2 and 0.4%) boosted the antioxidant properties and pasta's textural and sensory aspects, including color and flavor (Armellini et al. 2018). Saffron in yogurt lowered milk fat content, elevated yellowness and chroma, and diminished redness, hue angle, whiteness index, and lightness due to the presence of carotenoids, antioxidant compounds that extended the lifetime of the beginning cultures (Gaglio et al. 2019).

The function of two different saffron extract concentrations in wheat flour cookies on quality measures was investigated (Bhat et al. 2018). According to the findings, the saffron‐infused cookies retained significantly better‐quality features for up to 6 months of storage with no discernible loss. Since antiquity, the bulk of saffron output has been and remains to be used in cooking worldwide. Chefs and saffron experts have compared its scent to honey with metallic undertones. India, Iran, Spain, and other nations utilize saffron as a rice condiment. It is a common ingredient in many Spanish cuisines, including the rice‐based Paella Valenciana and the fish‐based zarzuela (Arshad et al. 2025).

Additionally, saffron is used in saffron cake, a peppery fish soup, Italian Milanese risotto, and French bouillabaisse. Saffron is used in the national dish of Iran, chelow kabab. Saffron is used in Indian cuisine's biryanis, which are traditional rice‐based dishes. Additionally, it is a component of several candies like Gulab jamun and kulfi (Mzabri et al. 2019). In Morocco, saffron is used in various traditional recipes, such as koftas (meatballs with tomatoes) or Mrouzia. It is also substituted for mint in tea (a sweet‐salty dish made from mutton or dill). Saffron is a key component of the chermoula herb mixture, giving many Moroccan recipes their signature flavor (Modaghegh et al. 2008).

### 4.3 Coloring Power

**FIGURE 4:**
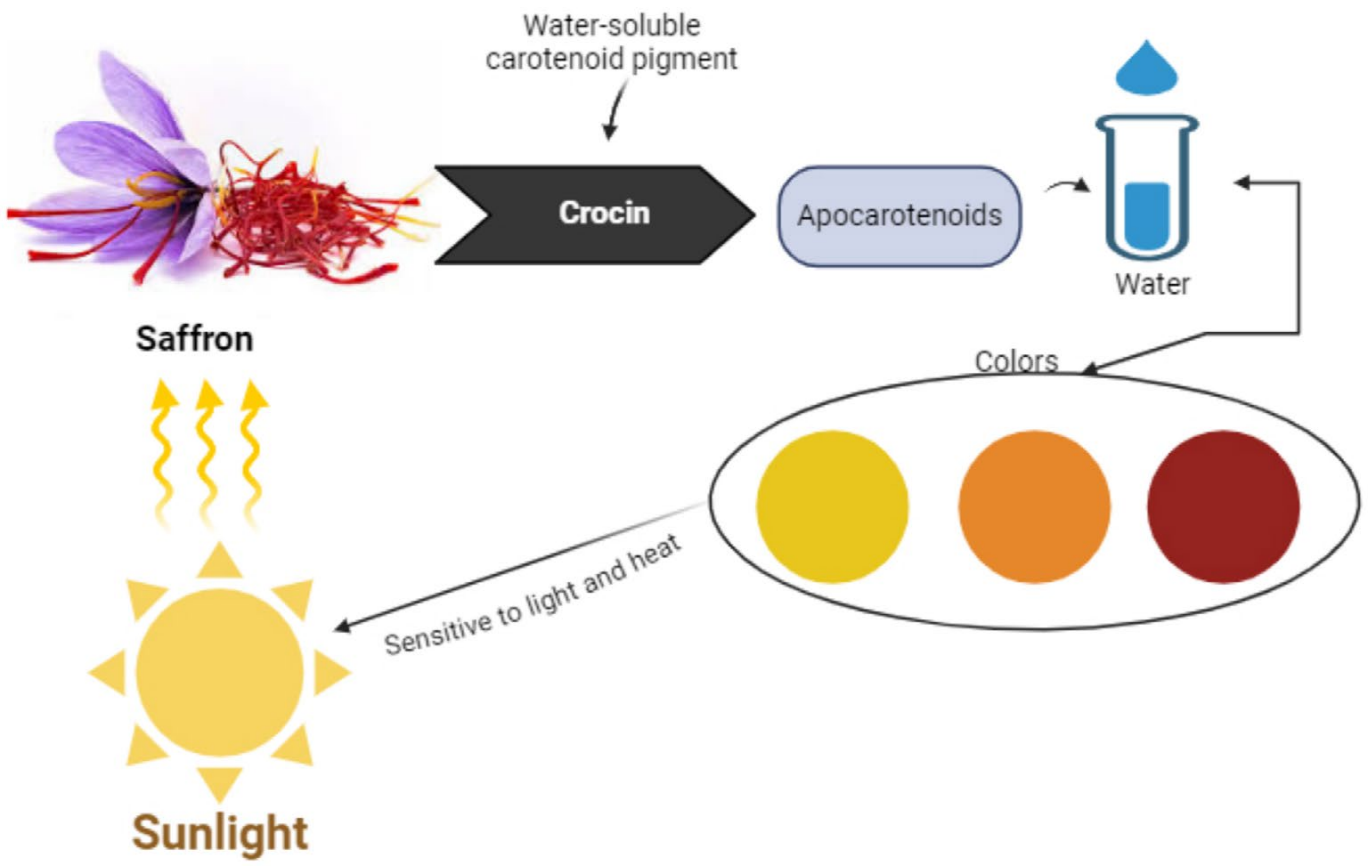
Crocin mechanism of action in saffron and its sensitivity to light and heat.

Synthetic food dyes have been banned in several countries due to their detrimental effects, and natural hues are now being used again. Because of the high absorption of crocin in water, saffron, as a replacement color, benefits the agro‐food sector. Thus, butter, pasta, cheeses, and even oleomargarine have all been dyed using the potent dyeing ability of saffron, which is also useful in cosmetics (Figure 4; Ramadan et al. 2012).

Saffron is a golden yellow color that is used in textiles and painting. Despite being in an acidic or alkaline environment, saffron solutions typically remain stable. Dicarboxylic acids, esters, nitrogen compounds, and crocin's pKa (acid dissociation constant) are all responsible for this feature. Saffron buffers slow down cellulose oxidation. Buddhist monks' clothing, as well as silk, wool, or oriental carpets, are still dyed with saffron today. Compared to some synthetic colors, natural dyes offer superior biodegradability, environmental tolerance, reduced toxicity, and are less allergenic (Mzabri et al. 2019).

## 5 Saffron Byproducts

They can be utilized as active ingredients in nutritional supplements, innovative food items such as juices, and cosmetic formulations, and as a possible natural source of anthocyanins for use in food and biomedical applications (Tuberoso et al. 2016; Moratalla‐López et al. 2019). Saffron tepal extracts can be used as a natural preservative in Pacific white shrimp stored on ice for 9 days, since they slow down melanosis, microbial proliferation, and lipid oxidation (Abbasvali et al. 2016).

According to Ahmadi Shadmehri et al. (2019), saffron petals extract displayed antibacterial activity against Gram‐positive bacteria *S. aureus* and Gram‐negative bacteria *Escherichia coli* . The entire blossom is delicious and has a flowery scent with hints of honey. Usage in vegan and vegetarian cuisine and for plating decoration is made possible by these intriguing features (Marchioni et al. 2019). However, flowers have a relatively limited shelf life after harvesting. Thus, drying at temperatures between 70°C and 90°C utilizing hot air convection maintains the highest amount of flavonols and anthocyanins (Serrano‐Díaz et al. 2013). Tepals, anthers, tunics, corms, and leaves have all been evaluated. Anthers are high in unsaturated long‐chain fatty acids and may be used to treat ulcerative colitis. The organic agent saffron petal has an impact in agriculture (Astarei Ali et al. 2006; Chichiriccò et al. 2019).

According to phytochemical studies, saffron petals include flavonoids and anthocyanins, which have positive benefits as auxiliary substances (Zeka et al. 2015). According to Mzabri et al. (2019) saffron lessens erythema, but more significantly, applying a mixture containing 3% extract on human skin may be helpful in the treatment of melanoma. According to research by Golmohammadzadeh et al. (2010) homosalate, an organic component found in several sunscreens, has a much lower sun protection value than 8% saffron lotion. Saffron petals are used as an antispasmodic, stomachic, anxiety‐curing, anticancer, and antidepressant in traditional medicine. It can be employed in various medical sectors per its economic features, phytochemical components, and conventional applications (Mortazavi et al. 2012).

## 6 Pharmacological Properties of Saffron

**FIGURE 5:**
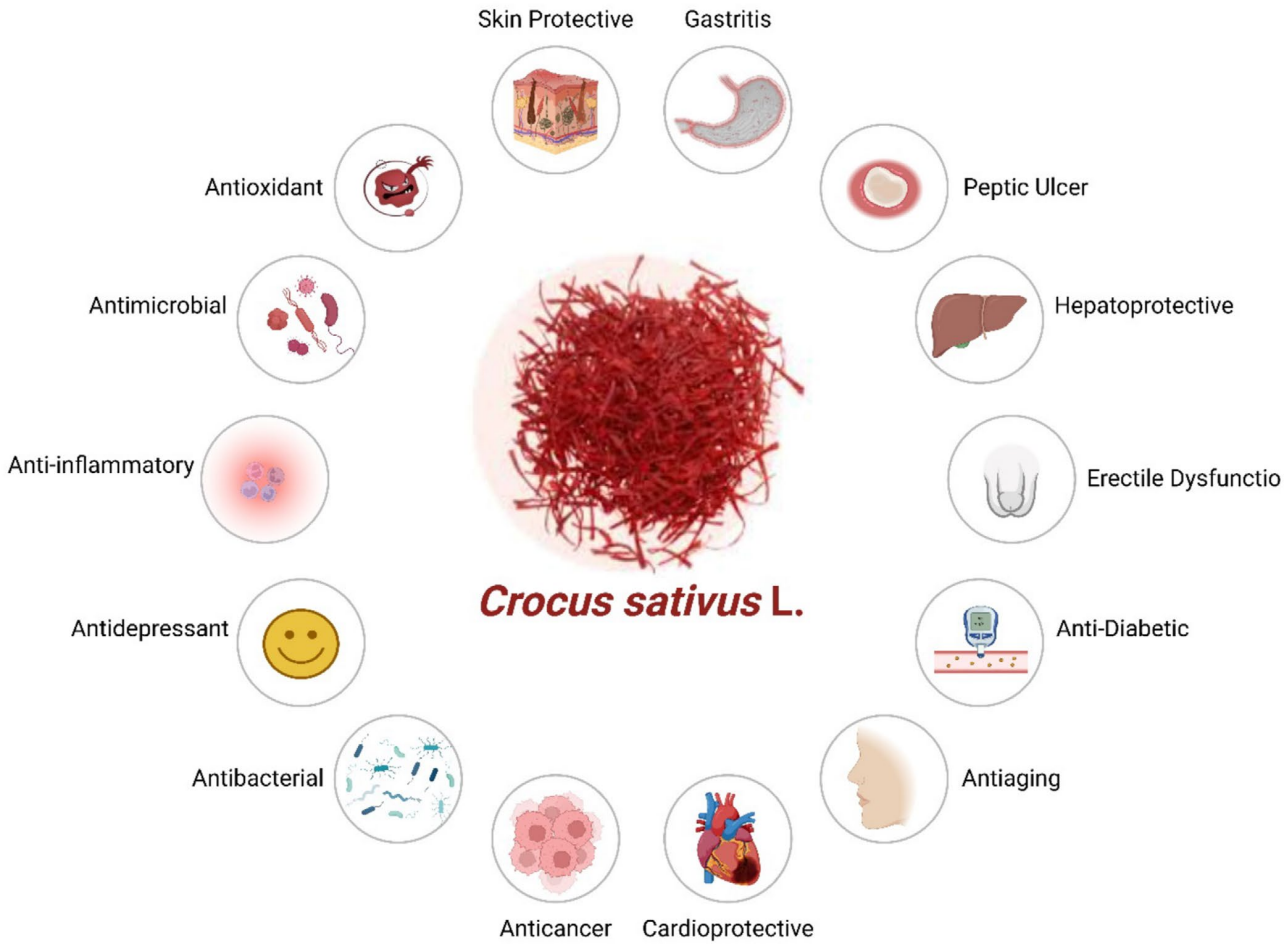
Health perspectives of saffron.

Saffron and its primary bioactive derivatives have been widely used in traditional medicine due to their numerous therapeutic properties, including their ability to protect against ischemia as well as their anticonvulsant, antidepressant, anxiolytic, hypolipidemic, anti‐atherogenic, antihypertensive, antidiabetic, and anticancer properties (Figure 5; Hosseini et al. 2018).

Crocetin has been linked to various modes of action, including an increase in oxygen transport during shock, a decrease in pro‐inflammatory chemicals, defense against oxidative stress, and the activation of death in cancer cells. However, it is important to note that while these findings are promising, more research is needed to fully understand the effects and mechanisms of action of saffron and its derivatives and to establish their safety and efficacy as therapeutic agents (Boskabady et al. 2010). Additionally, saffron and its derivatives have been shown to have various modes of action, such as blocking muscarinic receptors, stimulating beta‐2 receptors, and inhibiting histamine (H1) receptors (Hashemi and Hosseinzadeh 2019).

The anti‐inflammatory, antioxidant, and antiapoptotic properties of saffron's bioactive components are thought to play a role in its medicinal effects. These properties, especially their antioxidant capabilities, have garnered much interest for their potential impact on human health. Safranal and crocin have been shown to have antioxidant properties by scavenging free radicals (Hosseinzadeh and Younesi 2002). The exact mode of action of these carotenoids is not yet fully understood, but it is an active area of research (Moallem et al. 2014). However, it is important to note that while these findings are promising, more research is needed to fully understand the effects and mechanisms of action of saffron and its derivatives and to establish their safety and efficacy as therapeutic agents (Korani et al. 2019).

Saffron has been shown to alter the expression of genes related to the redox system of cells and inhibit the synthesis of DNA, RNA, and proteins, reduce telomerase activity, boost the pro‐apoptotic effect in cancerous cells, shift the stress marker genes in the endoplasmic reticulum system, modify the epigenetics, and form a strong union of crocetin with tRNA (Akbarpoor et al. 2020). Saffron's anti‐inflammatory properties are thought to be brought on by its capacity to scavenge free radicals and counteract the release of ROS and cytokines that cause inflammatory conditions. These findings suggest that saffron may have a range of potential therapeutic effects and that more research is needed to understand its effects and mechanisms of action fully (Table 4; Poma et al. 2012).

**TABLE 4:** Saffron effects and mode of action against diseases.

Disease	Effects	Mode of action	References
Depression	It has been demonstrated that saffron can lessen the symptoms of mild to moderate depression, with results that are on par with those of fluoxetine and other traditional antidepressants	By preventing serotonin from being reabsorbed in the brain, saffron raises serotonin levels, elevating mood and lessening symptoms of depression. Additionally, it impacts dopamine and norepinephrine levels, further increasing emotional well‐being	Lopresti and Drummond (2014)
Alzheimer's disease	Saffron increases memory and cognitive function while decreasing the progression of Alzheimer's disease	Saffron protects neurons from oxidative stress and inhibits the development of amyloid plaques, a characteristic of Alzheimer's disease. Additionally, it lessens neuroinflammation, which stops additional neuronal damage	D'Onofrio et al. (2021)
Parkinson's disease	By shielding neurons from harm, saffron may be able to delay the disease's course	Dopaminergic neurons get protected by saffron against oxidative damage and inflammation, two important aspects of Parkinson's disease pathogenesis. It also lessens the brain's oxidative stress, lowering the neurodegeneration chance	Hu et al. 2023; Inoue et al. (2021)
Cancer	Saffron stops the growth of cancer cells, triggers apoptosis, and stops them from spreading	Saffron inhibits the development of cancer cells by activating apoptotic pathways in cancer cells and causing cell death. Moreover, it prevents the development of new blood vessels, which is essential for the growth of tumors	Samarghandian and Borji (2014)
CVD diseases	Saffron lowers blood pressure, inflammation, and cholesterol to promote heart health	Saffron decreases LDL cholesterol and triglycerides while boosting HDL cholesterol. Additionally, it lessens blood vessel oxidative damage, which aids in the prevention of atherosclerosis. Additionally, crocetin aids in relaxing blood arteries, hence decreasing blood pressure	Su et al. (2021)
Diabetes	Saffron increases insulin sensitivity and decreases blood sugar	Saffron's crocin increases insulin's effectiveness and improves glucose metabolism. It also lessens inflammation, exacerbating insulin resistance and oxidative stress in pancreatic cells, enhancing insulin production	Sani et al. (2022)
Obesity	Saffron aids in weight management, decreases snacking, and regulates appetite	Saffron reduces serotonin levels, which in turn lessens cravings and emotional eating. Additionally, it lessens adipose tissue inflammation, which is linked to obesity	Mashmoul et al. (2013)
Premenstrual syndrome (PMS)	Premenstrual syndrome (PMS) symptoms such as mood swings, agitation, and pain are reduced with saffron	Saffron reduces PMS symptoms related to mood by balancing serotonin levels. Its anti‐inflammatory qualities may also help to ease menstruation pain	Agha‐Hosseini et al. (2008)
Sexual dysfunction	Saffron increases women's sexual satisfaction and helps men's libido and erectile function	Saffron's crocin enhances endothelial function, resulting in increased vaginal blood flow. It also boosts libido by controlling dopamine and serotonin levels	Ranjbar and Ashrafizaveh (2019)
Age‐related macular degeneration	Saffron increases visual acuity and protects against retinal degeneration	Saffron's crocin increases blood flow to the retina and inhibits the death of photoreceptor cells. Additionally, it protects retinal cells from age‐related macular degeneration (AMD) by lowering oxidative stress	Broadhead et al. (2019)
Gastric ulcer and peptic ulcer	Saffron helps gastric ulcers heal and shields the lining of the stomach	Saffron lowers inflammation and oxidative damage to the stomach lining and stimulates the production of mucus, which shields the lining from acid	Kianbakht and Mozafari (2009)
Hypertension	Saffron enhances vascular health and reduces blood pressure	Crocetin in saffron enhances the relaxation of blood vessels, which decreases blood pressure and prevents oxidative damage in blood vessels, consequently increasing endothelial function	Hooshmand‐Moghadam et al. (2021)

The anti‐inflammatory properties of saffron may result from the control of genes that control the secretion of pro‐inflammatory cytokines from glial cells, such as interleukin (IL)‐6, IL‐1, and IL‐2; (ii) preventing tumor necrosis factor (TNF)−, which is generated by microglial cells and causes DNA breakage, hence decreasing (Morya et al. 2024; Ikram et al. 2023). In a central cerebral artery occlusion model, crocin treatment inhibits blood–brain barrier (BBB) rupture by partially blocking the changes in tight junctions brought on by ischemia. When inflammatory cells and cytokines are absorbed into the neuronal environment, which might result in neuronal injury, the BBB must function with integrity (Zhang et al. 2017).

### 6.1 Antioxidant

Saffron has been investigated as a significant antioxidant agent because of the unusual chemical makeup of crocin (Srivastava et al. 2010). Because of its antioxidant capabilities, crocin was also the subject of a bleaching assay. One research study used methanol and water as solvents to compare the antioxidant activity of tomatoes, carrots, and saffron stigmas (Bathaie et al. 2011).

The antioxidant properties of saffron were also assessed, and safranals were shown to have lower activity than BHT and Trolox, but crocetin displayed equivalent values. Dimethyl‐antioxidant crocetin's ability was discovered to be dose‐dependent, with a peak value of 40 μg/mL. In contrast, saffron's synergistic effect made it appear to be a more effective antioxidant agent (Kanakis et al. 2007). According to Assimopoulou et al. (2005), crocin has a more substantial scavenging effect than safranal at 50% for 500 ppm solutions and 65% for 1000 ppm solutions. The hydrogen‐donating capacity toward the DPPH radical is thought to be responsible for the scavenging feature. The cells are protected from oxidative stress by the saffron‐related chemicals' scavenging of free radicals (Amarowicz and Pegg 2019).

In addition to reducing the peroxidation of lipids caused by ROS in hepatocytes, crocin has also proven successful in sperm cryoconservation, making it ideal for neurological illnesses. Malondialdehyde (MDA) production as a lipid peroxidation index brought on by ROS has also been shown to be reduced by crocin (Mehri et al. 2015; Uttara et al. 2009).

### 6.2 Antimicrobial

Microbes are everywhere and use a variety of cellular and molecular routes to adapt to changing environments, including those that may be contaminated with antibiotics. When using antibiotics, side effects and drug resistance have been key concerns. Scientists from all over the world are now searching for novel antimicrobial drugs with a good balance of safety and efficacy as a result of this. Food poisoning is one of the frequent health problems brought on by consuming foods tainted with dangerous bacteria (Wani et al. 2022).

We can avoid foodborne illness by using antimicrobial agents or certain preservatives to stop bacteria development. At a dosage of 1000 mg/mL with a zone of inhibition spanning from 12 to 22 mm, the methanolic extract of the saffron petal has demonstrated antibacterial efficacy against *E. coli, Bacillus cereus, S. aureus*, and *Shigella dysenteriae* . However, the activity is somewhat reduced when utilizing solvents like water and chloroform. Several researchers have also reported the antifungal activities of safranal and crocin, produced from saffron (Carradori et al. 2016).

### 6.3 Anti‐Inflammatory and Antinociceptive

Saffron's ability to stop diazinon (DZN)‐induced increases in inflammation, oxidative stress, and neuronal damage has been tested. IP delivery of aqueous saffron extract and vitamin E was utilized. Red blood cells' (RBCs') ability to cholinesterase was shown to be inhibited by DZN. In contrast, vitamin E or saffron did not affect this action. DZN all markedly elevated inflammation, oxidative stress, and neural damage markers. The saffron extract lessened the impact of DZN on the levels of these biomarkers (Wani et al. 2022).

The antinociceptive and anti‐inflammatory properties of the stigma and petals of *C. sativus* have already been investigated in aqueous and ethanolic extracts. A hot plate procedure was conducted to gauge the antinociceptive effects. To examine the effectiveness of extracts against acute inflammation, mice with xylene‐induced ear edema were employed. The efficacy of the extract's anti‐inflammatory properties was tested using formalin‐induced edema in the rat paw. When given intraperitoneally, neither extract had any antinociceptive effects on mice. The extracts demonstrated antinociceptive efficacy against acetic acid‐induced writhing. Extracts from both ethanolic and aqueous media have an anti‐inflammatory impact on chronic inflammation. The presence of flavonoids, tannins, anthocyanins, alkaloids, and saponins may be the cause of the antinociceptive and acute and chronic anti‐inflammatory effects that saffron stigma and petal extracts showed in a chemically induced pain test (Srivastava et al. 2010; Erfanparast et al. 2015).

### 6.4 Anticonvulsant

Pentylenetetrazole (PTZ) and maximum electroshock seizure (MES) tests were used to assess the anticonvulsant activity of saffron in mice using both aqueous and ethanolic preparations. PTZ failed to provide 100% mortality protection in the test, although it did show a delayed onset of tonic convulsions. Both aqueous and ethanolic extracts were found to reduce the tonic seizure duration in MES testing. The anticonvulsant properties of safranal and crocin have been studied in rats with PTZ‐induced convulsions (Rajabian et al. 2019).

Safranal has been shown to shorten the duration of seizures, postpone the onset of tonic convulsions, and shield mice from death. At 400 and 800 mg/kg doses, saffron showed significant dose‐dependent antiepileptic efficacy in a PTZ‐induced seizure paradigm. Safranal has been reported to lessen the length of PTZ‐induced seizures in mice and delay the beginning of tonic convulsions when administered intraperitoneally (Hosseinzadeh and Khosravan 2002; Wani et al. 2022).

### 6.5 Antidepressant

One in five people may experience symptoms of depression at some point in their lifetime, according to statistics. Depression is a commonly diagnosed condition in psychological diseases and mental illnesses. Instead of a health issue that might require medical attention, depression was frequently seen as a sign of weakness (Burić et al. 2016).

Saffron increases oxidative stress levels and weakens antioxidant defenses by lowering antioxidant enzymes such as superoxide dismutase (SOD), catalase (CAT), and glutathione peroxidase and increasing depressive‐causing oxidative stress indicators such as malondialdehyde (MDA). In liver tissue, safranal and crocin boosted CAT activity, while all three of saffron's active ingredients raised SOD levels and glutathione availability (Razak et al. 2017).

Serotonergic effects on depression could vary. Neurotransmitters with chemical qualities like norepinephrine, dopamine, and serotonin are key players in depression. Crocin has demonstrated antidepressant activity. According to studies, crocin affected the serotonin mechanisms by having an antagonistic action on the receptor site, which increased serotonin absorption. Crocin was found to have an affinity for the 5‐HT2c family of receptors, a nonselective serotonin (5‐HT) receptor (Kang et al. 2012).

Additionally, saffron's effects on serotonin availability can lessen premenstrual symptoms. According to research, crocin alters serotonergic systems by acting antagonistically on that receptor, causing a boost in serotonin absorption. Crocin was found to have an affinity for the 5HT2c family of receptors, a nonselective serotonin (5‐HT) receptor. Additionally, saffron's effects on premenstrual symptoms and serotonin availability can be lessened (Pirdadeh Beiranvanda et al. 2015).

### 6.6 Antibacterial

Saffron improves the immune system's ability to fight against infections and diseases brought on by bacteria. Saffron contains carotenoids that boost lymphocyte reactivity to mitogens, natural killer cell (NK‐cell) activity, immune cell protection from the immune cells' bactericidal creation of reactive species, and increased total white blood cells in HIV patients. Salmonella‐related food contamination was prevented by the bactericidal actions of safranal and crocin (Pintado et al. 2011). When used as a natural color for garments and textiles, saffron petal proved an efficient antibacterial against *Pseudomonas aeruginosa* , *E. coli* , and *S. aureus* (Ghaheh et al. 2014).

The callus and stigma of the saffron plant both exhibited variable factors of inhibitory effect on pathogenic bacterial strains in terms of their antibacterial activity. For instance, saffron stigma extract was superior to saffron callus extract in suppressing Shigella flexneri at the lowest concentration, 400 g mL^−1^ (Parray et al. 2015). Various saffron parts can be used to make ethyl acetate extracts that are effective against fungi and bacteria such as *Staphylococcus epidermidis*, *Micrococcus luteus*, *S. aureus*, and *E. coli* (*Aspergillus niger*, *Candida albicans* and *Cladosporium* sp.) (Hosseini et al. 2018).

### 6.7 Antitumor and Anticancer

Saffron can be a component of chemotherapy prevention therapy. Saffron has antitumor and anticancer functions, as evidenced by its ability to inhibit the creation of DNA and RNA but not protein, remove free radicals, participate in converting carotenoids to retinoids, and induce lectin‐mediated interactions. Crocin, according to some views, is the principal anticancer component of saffron. According to research, mice administered crocin were fully tumor‐free, but animals that were given a DNA immunization alone acquired around 66.7% and 33.3% of tumors, respectively (Bolhassani et al. 2014).

Safranal also prevented the expansion of the HeLa cell line, the proliferation of the MDA‐MB‐231 and MCF‐7 cell lines, and the reduction of several biochemical indications of diazinon toxicity. Crocetin has the potential to be an anticancer medicine since it inhibits the production of nucleic acids, boosts the antioxidant system, induces apoptosis, and blocks growth factor signaling pathways. Because liposomes may swiftly penetrate tumor sites from blood, saffron can be combined with liposomes to boost their anticancer effect (Parray et al. 2015).

### 6.8 Cardioprotective

A blood clot or the storage of fat plaque in the walls of arteries can restrict blood flow to the heart, causing cardiovascular disease (CVD), a general term for conditions affecting the heart and blood vessels (atherosclerosis). CVD can take many different forms: (1) coronary heart disease, often known as a heart attack, is a condition when a blood clot blocks blood flow to the heart and may result in angina (chest pain); (2) stroke, sometimes known as an ischemic stroke, is a medical disorder in which the brain is harmed or dies as a result of a blockage that prevents blood flow to the brain; (3) heart failure or congestive heart failure is characterized by an inadequate supply of oxygen‐rich blood to all body parts, which causes the heart to pump less efficiently than it should; (4) arrhythmia, a condition in which the heart beats abnormally (too slowly, too quickly, or irregularly) due to the electrical characteristics of the heart; (5) issues with the heart's valves, where the valve may not close properly or operate abnormally, as in mitral valve prolapse and stenosis, when the valve may not open wide enough to allow blood to flow; (6) (Razak et al. 2017; Smith Jr et al. 2001).

According to reports, saffron can prevent myocardium damage from isoproterenol. Saffron may be cardioprotective at all levels by preserving hemodynamics and maintaining structural integrity, left ventricular functions, and boosting antioxidant status. Lycopene, a flavonoid which is an antioxidant found in saffron tea, can lower the risk of cardiovascular disorders (Pintado et al. 2011). Saffron's electrical conductivity impact, which has a depressive effect on AV nodal rate‐dependent features, was demonstrated in an in vitro investigation. When used in high doses, saffron may prevent arrhythmia by slowing the electrical conduction velocity in the atrium and the ventricle. It was proposed that crocetin and crocin address endothelial dysfunction, particularly issues with aortic contraction, also known to have a hypotensive impact, by activating various processes in the vasoconstriction pathway in hypertension. Inhibiting pancreatic lipase causes saffron to be hypolipidemic and can lower triglyceride, low‐density lipoprotein cholesterol, total cholesterol, and very low‐density lipoprotein cholesterol levels in the blood. This increases the decrease of CVD risk in conditions like hypertriglyceridemia, atherosclerosis, and hypercholesterolemia (Sheng et al. 2006).

### 6.9 Antiaging

Age‐related macular degeneration (AMD) is presently regarded as the most known ocular degenerative disease to cause irreversible vision loss in affluent countries (Wong et al. 2014). According to studies, saffron helps prevent retinal degeneration in bright continuous light exposure rat models, an experimental model for AMD (Di Marco et al. 2014).

Saffron's antioxidant qualities and role in the regulation of genes that regulate glial cells' release of pro‐inflammatory cytokines were considered to be responsible for this neuroprotective impact (Natoli et al. 2010). In one placebo‐controlled study of AMD patients, saffron therapy (20 mg/day) was observed to enhance focal electroretinogram (fERG) results (Falsini et al. 2010).

Furthermore, these patients' 14‐month follow‐up showed that continued saffron medication improved their fERG and visual acuity metrics (Piccardi et al. 2012). However, randomized research found that saffron supplementation slightly enhanced visual performance in AMD patients. A longer duration of saffron therapy is probably necessary if the progression of such a chronic condition is not greatly retarded (Broadhead et al. 2019).

### 6.10 Antidiabetic

Diabetes mellitus (DM) is a metabolic illness that develops in the pancreas as it cannot create enough insulin for the body, or when it cannot utilize it effectively in some situations. Uncontrolled DM can result in hyperglycemia or high blood sugar levels, which, over time, can seriously harm the body's systems. According to the World Health Organization (WHO), there were 108 million people with diabetes in 1980 and 422 million in 2014. The WHO predicts that by 2030, DM will overtake heart attacks, strokes, liver failure, and kidney failure to become the 7th leading cause of death. DM can come in three forms (Vieira‐Potter et al. 2016).

Type 1 (T1DM), or juvenile, insulin‐dependent, or childhood‐onset diabetes, is characterized by a deficiency in the body's ability to produce insulin due to pancreatic‐cell failure. Type 2 (T2DM), also known as adult‐onset diabetes, is non‐insulin‐dependent and is characterized by a progressive decline in cell function and chronic insulin resistance. Saffron has been studied as a potential diabetes treatment option. Crocin, crocetin, and safranal are the principal antidiabetic components, and they have insulin‐sensitizing effects but have no detectable effect on blood serum concentrations of glutamic oxaloacetic transaminase (SGOT), glutamic‐pyruvic transaminase (SGPT), or creatinine (Kianbakht and Hajiaghaee 2011).

The primary theories for how saffron inhibits free radical chain reactions are that it can upregulate mitochondrial antioxidant genes and regulate antioxidant gene expression, which reduces the formation of oxygen radicals in the mitochondria. According to the results, saffron increased insulin sensitivity in glucose metabolism, preventing extra glucose buildup in the blood and activating 5′‐AMP‐activated protein kinase (AMPK), accelerating glucose uptake in skeletal muscle cells. Additionally, studies show that saffron supplementation improves the lipid profile, liver, and kidney function, as well as serum insulin and blood glucose levels in diabetic rats. In addition, saffron is said to boost SOD and glutathione content (GSH), which aid in lowering blood sugar levels and promoting pancreas regeneration. Saffron also possesses antioxidant qualities that can reduce oxidative stress and hyperglycemia, which may help treat diabetic encephalopathy (Samarghandian et al. 2014).

### 6.11 Hepatoprotective

The findings of animal studies suggest that saffron and crocetin may have hepatoprotective benefits against liver damage induced by toxic substances such as carbon tetrachloride and aflatoxin B1. However, results are limited to animal studies, and more research is needed to determine the safety and effectiveness of saffron and crocetin supplementation in protecting the human liver (Omidi et al. 2014).

Aspartate aminotransferase, alanine aminotransferase, gamma‐glutamyl transpeptidase, lactate dehydrogenase, glutathione S‐transferase (GST), hepatic glutathione (GSH), and glutathione peroxidase (GSH‐Px) levels as serum marker enzymes, as well as glutathione peroxidase (GSH). After data analysis, it was proposed that crocetin decreased the development of AFB1‐DNA adducts. Crocetin's beneficial effects on AFB1 hepatotoxicity may also be attributable to the hepatic tissues' defense mechanisms, which increased cytosolic GSH and the activities of GST and GSH‐Px (Khorasani et al. 2008).

The results of these studies suggest that saffron extract and its components (crocin and safranal) may protect against liver damage induced by toxic substances such as aluminum chloride and diazinon. However, these findings are limited to animal studies, and more research is needed to determine the safety and effectiveness of saffron supplementation in protecting the human liver. As with any potential treatment, consulting a qualified healthcare professional for advice is important. As a natural treatment, saffron can prevent hepatotoxicity due to its benefits for the liver's enzymes, antioxidant actions, and maintenance of the hepatic cell membrane. So, it would appear that saffron can effectively eliminate harmful elements that lead to liver damage (Mousavi et al. 2022; Hasani et al. 2021).

### 6.12 Gastritis and Peptic Ulcer

Crocins contain gastroprotective properties in ethanol‐induced stomach damage. Levels of gastric juice rose with crocin pretreatment. IL‐6, TNF‐, and prostaglandin E2 (PGE2) levels increased in the mucosa. Heat shock protein 70 mRNA and protein levels dropped, as did myeloperoxidase activity. Crocin therapy restored the mucosal levels of malondialdehyde, glutathione, and SOD activity. Through the downregulation of cytochrome c and caspase‐3 expression, a reduction in caspase‐3 activity, and the mitigation of DNA fragmentation, crocin pretreatment lessens the ethanol‐induced mucosal apoptosis (Ashktorab et al. 2019).

El‐Maraghy et al. (2015) found that crocin exhibits anti‐inflammatory, anti‐apoptotic, anti‐oxidative, and mucin‐secretagogue mechanisms that protect rat stomach mucosa from ethanol‐induced damage. These mechanisms are likely mediated by enhanced PGE2 release. Saffron's antioxidant qualities could help to prevent the deterioration of the stomach mucosa by increasing glutathione levels and lowering lipid peroxidation. According to Inoue et al. (2005), saffron can prevent ulcers from being brought on by stress and histamines. In a similar vein, Al‐Mofleh demonstrated the potent anti‐secretory and anti‐ulcer properties of saffron. These findings need to be further investigated through clinical trials to compare saffron to other medicines for treating peptic ulcers (Ashktorab et al. 2019).

### 6.13 Skin Protective

Saffron has been used as one of the ancient Persian medicines to shine, brighten the skin, and lessen black pigments, acne, dark circles under the eyes, and pimples. Crocin may act as an activator for DNA repair enzymes or an inhibitor of DNA damage‐causing substances. Additionally, saffron can absorb UV radiation from the sun due to its antisolar action (Jafari et al. 2020).

Moshiri et al. (2015) investigated 3% dried saffron extract by inoculating it in o/w lotion, cream, and face powder formulations on people between 18 and 28. They said saffron could unmistakably brighten the skin. They claimed that crocin and crocetin found in saffron are to blame for these effects. The antioxidant properties of crocin and crocetin may be responsible for saffron's shining and depigmenting effects. Saffron has been shown to reduce melanin formation. As a consequence, it works wonderfully for skin lightening. The combination comprising *C. sativus* extract exhibited a considerable depigmenting and antiarrhythmic effect on human skin (Das et al. 2010).

Melanocytes combine the two pigments, melanin and pheomelanin, which are brown‐black and red‐yellow, respectively, to generate melanin in the skin (Moshiri et al. 2015). Multiple oxidative processes regulated by different enzymes result in the process of melanogenesis. The primary initiator of this process is tyrosinase. Monoterpenoids, crocin, quercetin, kaempferol, and other phenolic substances found in *C. sativus* all display antioxidant action. These substances work by suppressing the function of tyrosinase to diminish skin melanin (De Leeuw et al. 2001).

## 7 Therapeutic Potential of Saffron

### 7.1 Alzheimer's Disease

The most prevalent ailment among seniors is Alzheimer's disease (AD). AD, a kind of dementia, can cause a slow decline in mental abilities that impairs one's capacity to carry out daily tasks. Patients with AD also experience personality and behavioral changes. Around the world, AD has already impacted 27 million people, and by 2050, that number is projected to rise to 86 million. Saffron has been proven to boost learning and memory skills, have a geno‐protective impact, and protect against oxidative stress induced by genotoxins because of the variety of bioactives present. Crocins are mono‐and diglycerides of crocetin (CRT) and acetylcholinesterase inhibitors for treating AD (Doecke et al. 2012).

Crocin not only enhanced memory formation but it also inhibited long‐term potentiation, which ethanol inhibits in vivo. LTP, a type of activity‐dependent synaptic plasticity, is thought to be the fundamental substrate of memory and understanding in the hippocampus. Crocin, unlike picrocrocin, can block ethanol's activities on NMDA receptors. Saffron inhibits amyloid formation in the human brain due to the positive action of crocin, which promotes protein stability (Razak et al. 2017).

### 7.2 Parkinson's Disease

The decline of projecting dopaminergic nerve fibers in the striatum triggered by the destruction of dopaminergic neurons in the substantia nigra is a hallmark of Parkinson's disease patients' progressive neuropathology (SN). Endogenous 6‐hydroxydopamine (6‐OHDA), a neurotoxin that selectively damages dopaminergic and noradrenergic neurons in the brain, has also been observed in Parkinson's patients (El Midaoui et al. 2022). It is worth noting that research utilizing a 6‐hydroxydopamine (6‐OHDA) rat model of Parkinson's disease found that crocetin protected substantia nigra neurons from 6‐OHDA's harmful effects by conserving reduced glutathione (GSH) and dopamine levels and reducing TBARs (Ahmad et al. 2005).

Crocins boost the memory capacities of the same animal type. Crocin's favorable benefits on memory in Parkinsonian rats, according to the scientists, were produced, at least in part, by a drop in TBARs and nitrite (NO_2_) values in the hippocampus. Crocetin treatment was found to lessen motor deficits and preserve dopaminergic neurons in a different animal model of Parkinson's disease induced by 1‐methyl‐4‐phenyl‐1,2,3,6‐tetrahydropyridine (MPTP), in addition to providing protection against mitochondrial dysfunction by obstructing the establishment of the mitochondrial permeability transition pore (mPTP) (Dong et al. 2020).

### 7.3 Erectile Dysfunction

As defined by the National Institutes of Health, erectile dysfunction (ED) is the failure to obtain or maintain a strong enough erection for sexual engagement. To put it another way, ED is a man's persistent or recurring inability to produce and keep a firm enough penile erection for sexual interaction. ED may be diagnosed using a variety of approaches. A new standard technique called the International Index of Erectile Function (IIEF) has been created to measure ED using a more precise multidimensional scale adaptable to changes in erectile function (EF). Nitric oxide (NO) is now acknowledged to be a crucial penile erection mediator as well as a messenger of smooth muscle relaxation. Currently, ED is regarded as a severe health concern, with 322 million cases expected by 2025 (Cirino et al. 2006).

Beginning with psychosexual and couples therapy for purely psychological ED and relationship issues, the treatment for ED is followed by lifestyle changes like weight loss, exercise, and quitting smoking. It concludes with testosterone supplementation for any related hypogonadism. These medications improve erections physiologically by boosting blood flow, altering hormone levels, and relaxing the smooth muscle of the corpus cavernosum. In the past, ginseng, saffron, nutmeg, ambrein, and cacao were used as aphrodisiacs (Melnyk and Marcone 2011).

According to studies, saffron significantly improves ED using the IIEF test and nocturnal penile tumescence (NPT), a less expensive alternative to PDE5‐Is. Saffron's crocin, which enhances mounting frequency (MF), EF, intromission frequency (IF), intromission latency (IL), decreased ejaculation latency (EL), and mount latency, is what causes this therapeutic effect (ML). Although some research indicated that saffron could treat sexual dysfunction, long‐term chronic effects have not been established (Modabbernia et al. 2012).

The antitussive benefit of safranal and crocin, two stigma and petal extracts from *C. sativus* , in guinea pigs that had previously received a nebulized treatment of 20% citric acid—the intraperitoneal injection of the extract and the agents. Only when saffron ethanolic extract and safranal were administered did the antitussive function identified by a decrease in the sum of coughs become apparent. Such an impact was absent in the ethanolic and aqueous extracts of petal and crocin (Hosseinzadeh and Ghenaati 2006). The relaxing effects of aqueous and ethanolic extracts of *C. sativus* and safranal on guineapig tracheal chains were also studied by (Boskabady et al. 2010). Safranal is at least partially to blame for the relaxing effect that was noticed by Wagner et al. (2000).

### 7.4 Pancreatic Disorders

A high‐fat diet is one of the main factors contributing to insulin resistance. Because of how crocetin affects lipid metabolism and has a hypolipidemic effect, it can have an impact on insulin resistance. Crocetin also increased the uptake and oxidation of non‐esterified fatty acids in the liver, sped up the removal of triglycerides from the blood, increased the activity of lipoprotein lipase in the liver, and decreased the buildup of harmful lipids (DAG and long‐chain acyl CoA) in the liver and muscle. To hasten lipid uptake and oxidation, peroxisome proliferator‐activated receptor‐regulated genes involved in hepatic lipid metabolism were altered (Sheng et al. 2008).

According to research, crocetin can treat a variety of abnormalities connected to a decline in insulin effectiveness. To determine the therapeutic benefits of crocetin on insulin resistance and pancreatic gland anomalies, dexamethasone, therefore, caused insulin resistance in a rat model. These mice had noticeably higher levels of serum insulin, free fatty acids (FFA), triglycerides (TG), and tumor necrosis factor (TNF−). Additionally, the function of the pancreatic islet beta cells was observed to improve and the hepatic glycogen content to decrease. The results show that crocetin could eliminate or decrease all aberrant items indicated after use (Xi et al. 2005).

In a subsequent study, Xi et al. (2005) examined the effects of crocetin on insulin resistance and the anomalies linked to it that are brought on by a high‐fructose diet. Some symptoms of a high‐fructose diet include insulin resistance, dyslipidemia, hyperinsulinemia, hypertension, increased tumor necrosis factor (TNF−), and decreased expression of adiponectin's protein and mRNA. Analysis of the variables following treatment demonstrated that crocetin has the power to reduce or even reverse the harmful effects of a high‐fructose diet. The anti‐hyperglycemic effects of tolbutamide, a common hypoglycemic medication, and ethanolic saffron extract were examined. They used alloxan to induce diabetes in rats to achieve their goal. The pancreatic islet cells of control and diabetic rats were studied histopathologically and immunohistochemically in addition to fasting blood glucose (FBG) levels. According to the study, the saffron extract has an anti‐hyperglycemic action that can benefit an injured pancreas (Rahbani et al. 2012).

### 7.5 Primary Dysmenorrhea

About 90% of women experience primary dysmenorrhea, which is characterized by lower abdominal cramps before or during the menstrual cycle without the presence of other disorders. A pilot RDBPCCT on 180 female students with primary dysmenorrhea aged 18 to 27 years was used to evaluate the effectiveness of saffron in the treatment of the condition. Three groups of people were randomly assigned to receive either a placebo, mefenamic acid, or herbal remedies (saffron, celery seed, and anise extract three times daily for 3 days) beginning at the first sign of bleeding or pain. Pain intensity and duration were monitored for 3 months. Compared to the placebo, both herbal and pharmaceutical medications effectively reduced menstrual pain (Luo et al. 2024; Nahid et al. 2009).

Another study by Pirdadeh Beiranvanda et al. (2015) looked into how the smell of saffron affected some gynecologic symptoms like PMS, dysmenorrhea, and irregular periods. For 20 min, the saffron odor was inhaled by 35 healthy, normal‐smelling women with regular menstrual periods. The enzyme immunoassay method was then used to determine the 17‐beta estradiol, testosterone, and cortisol amounts in saliva samples. For psychological testing, the State–Trait Anxiety Inventory (STAI) was utilized. Following exposure to the saffron scent in both the follicular and luteal phases, the result was a significant decrease in cortisol levels and increased estradiol levels. The STAI score also dropped in the saffron group's follicular and luteal stages. To treat PMS, dysmenorrhea, and irregular menstruation, the results of this RDBPCCT revealed that saffron odor had favorable physiological and psychological impacts. However, in this study, ethanol was used to dilute the scent of saffron so that test subjects could detect it. This suggests that such a solvent may be an effective treatment (Pirdadeh Beiranvanda et al. 2015).

## 8 Toxicity

For the most part, saffron is safe because it has been a food ingredient for many years. It was necessary to assess the toxicity and safety of saffron when used as medicine. Due to its notable nutritional benefits and medicinal characteristics, saffron has been used as a spice for thousands of years. A study on the toxicological effects of saffron and its components is crucial to determine the proper doses and their safety. The appropriate dosage must be used for saffron to be safe and effective. Since most studies are carried out in vitro and in vivo, it is difficult to identify the proper human dosages (Bostan et al. 2017).

Saffron extracts have historically been used to heal illnesses without adverse side effects. Numerous studies have been conducted to assess the feasible toxicity of saffron in experimental animal models more recently as a result of the use of saffron in clinical trials. No substantial alterations in the hematological and biochemical parameters indicating its toxicity have been discovered in any of these trials. Saffron in excessive dosages should be avoided while pregnant. Doses greater than 5 g, which are far higher than those consumed during meals, may stimulate the uterus and result in abortion (Wani et al. 2022). Saffron's toxicity and safety were investigated in a study that divided healthy participants into three groups and gave them daily 400 mg pills for 7 days. Saffron was discovered to alter several hematological and biochemical indicators; nevertheless, any modifications were deemed within normal ranges and did not suggest any clinical disorders (Modaghegh et al. 2008).

Another study by Wani et al. (2022) experimented on 32 pregnant mice to test the acute toxicity of saffron at doses of 500, 1000, and 2000 mg/kg/day for 3 weeks. The group acting as the control received the regular saline solution. The progeny's harmful effects, including nephrotoxicity and hepatotoxicity, were investigated. In a subacute evaluation, saffron was determined to be safe with no negative impact on the liver, although high doses given to moms could affect the offspring's kidneys. In animal tests, the mean fatal dosage (LD_50_) of saffron was 20.7 g/kg, while in vitro, it was 200 mg/mL. When the aqueous extracts of saffron and safranal are combined, the acute and subacute toxicity of safranal is significantly reduced. Daily 30 mg doses for depressive disorders in clinical trials did not differ in tolerance from placebo, although there were adverse effects such as vomiting, nausea, and headaches (Wani et al. 2022).

## 9 Conclusion and Future Trends

Saffron, a low‐toxicity medicinal plant, offers many potential health benefits due to its potent antioxidant properties, primarily attributed to bioactive constituents like crocetin, crocin, carotene, and safranal. These components effectively scavenge free radicals, making saffron a promising natural food additive. Both in vitro and in vivo studies confirm its antioxidant efficacy, comparable to carotenoid‐rich foods. This activity stems from the synergistic action of its bioactive components. Beyond its antioxidant capacity, saffron's therapeutic potential extends to various areas.

Future research should investigate its potential neuroprotective effects and its role in managing neurodegenerative diseases, examine its impact on cardiovascular health, explore its traditional use in treating respiratory ailments like bronchitis and asthma, investigate its potential role in regulating blood sugar levels and managing diabetes, further research its potential to alleviate stress, anxiety, and improve overall mood, and explore its potential anti‐inflammatory and pain‐relieving properties. Optimizing saffron's bioavailability through targeted formulations and investigating potential synergistic effects with other therapies are crucial areas for future research. Developing saffron‐based functional foods and exploring their impact on overall health and well‐being also represent promising avenues for future investigation.

## Author Contributions

**Tabussam Tufail:** project administration (equal), writing – original draft (equal). **Huma Badr ul Ain:** formal analysis (equal), investigation (equal). **Ali Ikram:** supervision (equal), validation (equal). **Muhammad Tayyab Arshad:** data curation (equal), writing – review and editing (equal). **Muhammed Adem Abdullahi:** project administration (equal), writing – original draft (equal).

## Ethics Statement

The authors have nothing to report.

## Consent

The authors have nothing to report.

## Conflicts of Interest

The authors declare no conflicts of interest.

## Data Availability

The data supporting this study's findings are available from the corresponding author upon reasonable request.
